# Veterinary Education in Relation to the Practice of Veterinary Medicine in New York State1A paper presented to the New York State Veterinary Medical Society, September, 1894.

**Published:** 1895-01

**Authors:** A. Liautard


### THE JOURNAL

OF

### COMPARATIVE MEDICINE AND VETERINARY ARCHIVES.

Vol. XVI. *JANUARY*, 1895. No. 1.

### VETERINARY EDUCATION IN RELATION TO THE PRACTICE OF VETERINARY MEDICINE IN NEW YORK STATE.[1]

BY PROFESSOR A. LIAUTARD, M.D., V.M.

Mr. President and Gentlemen: To be called at this moment before such an honorable gathering of members of the veterinary profession is an honor which to me comes to crown a period of thirty years engaged in behalf of the profession; and when I am asked to address you on the subject of veterinary education in relation to the regulation of the practice of veterinary medicine, you can appreciate how I realize the compliment and yet how regretful I must be of what I consider my inability to do justice to the subject. I hope, therefore, that while you will accept my warm thanks for the honor devolved upon me you will also receive my remarks with indulgence and with the assurance that if I err in my conclusions it will be with the firm belief that I am still speaking for what I consider a good cause.

The subject of "Legislative Regulation of the Practice of Veterinary Medicine in the State of New York" is one of the natural consequences of the well-deserved reputation and past history of the State, namely, that of being the *one* in the Union where higher education is the principal aim of her citizens. The *first*

[1] A paper presented to the New York State Veterinary Medical Society, September, 1894.

in almost everything, New York State has been the leader in witnessing the establishment of veterinary schools on comparatively solid foundations, even if these are the results of private undertakings; she was the *first* where, for higher standing of her veterinarians, her veterinary schools were seen to require a matriculation examination, and she was among the *first* in the endeavors which were made to obtain legislative action to regulate the practice of that branch of medicine. The first act in that direction was passed by the Legislature in Albany in 1884.

But these were only the first steps in the right direction. The profession has made great strides toward improvement since the day when the first veterinary school had opened her course in New York City in 1864; a daring step had been taken by the American Veterinary College when, in 1875, candidates to her degrees were requested to pass an examination before they could matriculate and enter her lecture-room, and though this examination continued to be made more severe and more difficult, little by little, they were and are yet inadequate to the requirements and to the qualifications of the educated veterinarian.

In former days, as many of you may remember, and up to the last year, the requirements of our veterinary colleges (I speak of those in this State) were an attendance of two courses of lectures. First, there were only short sessions of a winter course of four to four and one-half months; then a spring session was added, making the course six months; then came the two years' course of six months each; and now we find in the American Veterinary College a requirement of attendance at three winter sessions of six months each, which will be increased to eight months in a short while.

This result has been reached by degrees, as you see; and if we have arrived at this point, if among the successful veterinarians of this Excelsior State we can count many of the alumni of the schools of New York, if we can boast of the progress made by our profession, of the standing to which the veterinarian has reached, there is no doubt that it is due to the fact that our colleagues are possessed of higher education and are ambitious to increase their professional knowledge, reared in that direction as they were while at college, where they were admitted with more or less difficulty, and already in possession of a self-desire for more knowledge and a comprehension that *knowledge* and *education* are *inseparable*.

Does all this, however, mean that though we have so far advanced we have reached the limit of our needs? Our veterinarians are far better educated than they were some years ago; but does it mean that still higher education is unnecessary or would be superfluous?

Evidently no; and I am quite certain that the days are not far off when, in the United States, the standard of veterinary education will be that which has for years existed on the Continent and lately established in England, viz.: an obligatory attendance at college of four sessions of at least eight months each.

Of course, this will have to be reached by slow progress; but with it another condition will have imposed itself, viz.: *higher education before matriculation.*

Like in my studentship time, an ordinary good, though special, education was only required, and to-day a degree of bachelor of science or of arts is demanded by the French schools. The same will be demanded here—this, in fact, already exists. Several of the American Veterinary College Alumni had received the A.B. or B.S. before the D.V.S. was added to their names.

But how long has it taken for European schools to reach that point?

If Lyon and Alfort are a century old, and if it is only at the end of those hundred years that such facts have been accomplished, can it be expected that after an existence of only thirty years we can accomplish it?

The progress that has so far been made, as results of *required improved education*, are too evident for me to dwell upon.

The class of practitioners, of the young men who enter the profession, now are far superior to those of years gone by. The work that has been accomplished by the members of the veterinary profession has spoken by its results; greater public consideration is now granted to the veterinarians of to-day as compared with that given to the old self-made practitioner; legislative bodies are listening to our claims. The Regents of the University of the State are devoting some of their valuable attention to the subject of veterinary education and to the "Regulation of the Practice of Veterinary Medicine in the State of New York." From all this we realize that we are coming close to the period I was alluding to a moment ago.

As you are aware, a bill was presented at the last Legislature in relation to those important subjects.

Framed upon the medical bill now in force in the State, the bill recommended the creation of a board of veterinary examiners; specifications were presented as to the duties of that board, as to the qualifications of those who would be allowed to present themselves for examination before said board, and also as to the primary requirement—the essentials for matriculation and graduation from our veterinary colleges.

The principal object of the bill was higher education, and one that no one can object to it, myself least of all; but as long as we are now speaking on this subject, I hope you will allow me a few remarks on some of the requirements of the bill.

The creation of a board of examiners is one that I cannot strongly indorse. I have on previous occasions, and in various papers, pointed out the importance I attach to the existence of such board, and if I erred in calling for a national board, overlooking as I did the difficulties that exist in the creation of such a body through the fact of State rights, I am perfectly satisfied to see the plan started to-day in each individual State until the moment has come when the importance of the veterinary profession will have been proved so powerful as the guardian of our national agricultural wealth, the Federal Government will then take hold of it in the proper way and make it one of its national institutions, as it is in the Old World.

The conditions of admission to examination before the State Board form the most interesting part, however, of the bill to the future veterinarian and to the existing places of education.

Section 176 of the bill reads:

> The Regents shall admit to examination any candidate who . . . is more than twenty-one years of age, of good moral *character, has the general education required in all cases after July 1, 1896, preliminary to receiving a degree in veterinary medicine, not less than three full years*, and has received a degree as veterinarian from *some registered* veterinary school.

These requirements are all that we have been asking for; in fact, all that which imposes itself upon the veterinarians and upon veterinary schools of repute, that which must necessarily be carried out all through the country in a short time.

It means for us the complete indorsement of our remarks and of our propositions made in Chicago before the members of the First Veterinary Congress of America, held last year. It means all obligations for every one who shall intend to practise in *this State* to obtain certificate of qualification and of registration; it means no more imposition on the part of the easily obtained graduation, from any school, from any part of the world; it means death to quackery; it means the elevation of the veterinary profession on its proper pedestal.

And yet with all these great benefits obtained, with all these obligations which are going to make the veterinary profession of New York State what we are all wishing for, why do we have some objections to make?

The reason is simple, and, though it may appear personal on my part, I will not hesitate to mention it, feeling as I do that no one here will accuse me of personal feeling when the subject of veterinary elevation is at stake.

Gentlemen, years ago, when I began the hard work of starting the first veterinary school open in New York, I admitted to matriculation gentlemen who were earnest in their intentions, in their purposes, but whose education—well, I will say nothing of this; some stayed with us, worked hard and succeeded—others, well, they failed or retired to engage in other more suitable business, and so it went until 1875.

I do not mean, however, to carry the impression that all the students at the New York school at that day were inferior in their education. Some of them have made their mark in the profession—some of them are to-day among the foremost of our colleagues.

In 1875 the American Veterinary College opened its doors; the matriculation requirements were carefully considered and imposed in 1877, when *certificates of collegiate or academic education or a preliminary examination are demanded.*

Of what did that preliminary examination consist? It was not very severe, you may believe, but yet it proved a benefit—better students came to us.

In 1885 the requirements at the A. V. C. are made more stringent and better defined. "They demand a *good common school education*, *certified* by *college degree* or an examination on *reading*, *writing*, and *spelling*."

In 1889 a still more severe test is demanded, as the examination now will consist in an *essay* as test of *handwriting*, *orthography*, *grammar*, and *reading from* a *classical* work.

In 1890 no change is made, but the *better educated the candidate is the better will it be for his further studies.*

In 1894 the requirements are either a certificate or diploma of a college degree, or "certificate of an examination passed before a recognized college, or of having attended the highest class of a public school," or "an examination which will serve as a test in *reading*," *handwriting*, *orthography*, *grammar*, and *arithmetic*.

Gentlemen, it has taken us twenty years to reach this still low standard. We have progressed slowly, perhaps too slowly, some will say. I will answer, " Yes, but surely."

It is well enough for some of our friends to say that the requirements demanded by the Regents' bill are certainly very low, and that after all the examination on *arithmetic*, *elementary English*, *geography*, *spelling*, *United States history*, *English composition*, and *physics* cannot be considered as too severe a test for a candidate to the study of the immense field of comparative medicine.

I will grant that " the better educated, the better fitted." But are our young men prepared for this sudden raise in the requirements? Many possess those qualifications, no doubt, but how many do not? It may be proper to expect them from our city boys, but how about those who are in the country? It may not be difficult to find them among families well-to-do, but how about the ambitious boy who has by hard work—I was almost going to say hard labor—learned some of the requirements that you ask, has economized to see his way through the three years of college life . . . and who finds the college doors closed for the present, at least for several years anyhow, until he becomes possessed of your new reinforced obligations?

But let us suppose that we are in error, that the officers of the American Veterinary College have been wrong in working so carefully since their organization, and that the requirements of the bill of the Regents should be the only one which would allow our veterinary schools to grant degrees to veterinarians who would establish themselves afterward in the State. What would it mean? What would be the result?

Simply this: *Private schools, give up your charter and close your doors. Your existence of thirty years' teaching*, *gentlemen* of the *faculty*, *are no protection.* Your ungrateful work, your years of persistent activity, your sacrifices in behalf and for the love of your profession, must all be ignored. *Your schools will remain empty*—not because of your deficiency, perhaps, but of the severe requirements for which our young men had no opportunity and no time to prepare, and which have *chased them in the veterinary* halls of other States.

Do not believe, gentlemen, that I exaggerate. I have seen enough of this done in the past, and if it proves to be so when the requirements were so slowly, so gradually increased, what will it be when the new régime is re-enforced?

You may say that if the students from the State will go to graduate in other States they will have at their return to submit to the requirements of the Regents' bill before they can practise.

This is true. But how do you know that they will return? And what will be said of New York, the "Excelsior" State, whose veterinarians would then become alumni from other schools and graduates out of the State?

The alumni association of the American Veterinary College counts 200 gentlemen out of her 530 graduates, 200 who are from New York State and New York City, most of whom are practising in the State.

How many more hundred are likely to follow their example, and yet the life of our private schools, of those who were the nursery of the *American veterinary profession*, with their existence of hard and persistent devotion to her, a proud life, as it is in fact, is threatened, and her existence may be suddenly ended by a stroke of the pen of our Governor.

Gentlemen, no one in this building knows what the foundation and elevation of a place of instruction, and specially of a veterinary school, exacts, of work, of restless nights, of patience, of sacrifices of all kinds. Some imagine that it is all smooth road; that all there is needed is to organize, issue circulars, open, go on . . . or close. But if anyone entertains those ideas, let him be sure it will take but a very short time to satisfy him of his error.

Our schools as they have been since 1864 are private undertakings; their life, their improving curriculum, their success, their existence, have depended on the number of students which have come to them; they have not behind them the support of wealthy and well-known organizations—for these last, 100 students or no student may mean alike—for the private schools, no student or a limited number mean an arrest in their work, a lack of life support, the closing of their doors.

And then what? Is our State prepared to receive the future students (from 75 to 100 last year) who wait for admission to the schools? Is there another veterinary institution which is ready to admit them, with the same facilities, same opportunities for theoretical or practical work?

If there is, then the Regents are right. A school (I mean

State school) with everything that is necessary, with perfect organization, is what is wanted in our large States. When once such State institutions exist, private undertakings will scarcely have a right to existence; but until then do not cripple the efforts of the schools of the day, which, after all, have done so well and against which you cannot even reproach their slow growth as long as you have not granted an *iota* to their help.

But here, Mr. President and gentlemen, I have occupied your attention quite long enough. I have endeavored to show you how a higher degree of education is necessary for our future candidates to a veterinary degree; how with this desire in view the A. V. C. has little by little reached the point where she is to-day; I have tried to show you what might, what will be the result of too sudden and severe requirements upon the existence of our private schools in the State, and which in case of death cannot be replaced *immediately;* and it is in the behalf and for the welfare of these mothers of the American veterinary profession that I will close these remarks in recommending to the favorable consideration of the meeting the following conclusions, namely: that we indorse the passage of a bill recommending the establishment of a State examining veterinary board, in *which* the requirements for matriculation to any of our State veterinary schools, besides those including degrees of an academy, high school, etc., shall be a certificate from the Board of Regents of the University, based upon a graded scale in such a way that for the *year* 1895–96 the examination shall be on *English composition* (that is, spelling, writing, reading), *arithmetic and United States geography;* for the year 1896–97, *English composition*, *arithmetic*, *geography*, and *United States history;* for the year 1897–98 *English composition*, *arithmetic*, *geography*, *United States*, *history* and *elementary physics*, and in which the condition of admission to examination of a graduate before said board shall be a degree from a veterinary school or veterinary department of a university having at least a *three-years' course* of studies and *college attendance.*

---

The Department of Meat Inspection of the city of Philadelphia has just made its report, in which we note that 209 cattle were condemned, 109 for tuberculosis, two for actinomycosis, and the rest for other causes. One hundred and thirty-one sheep were also condemned.

